# Early myogenic responses to acute exercise before and after resistance training in young men

**DOI:** 10.14814/phy2.12511

**Published:** 2015-09-09

**Authors:** Marissa K Caldow, Emily E Thomas, Michael J Dale, Grant R Tomkinson, Jonathan D Buckley, David Cameron-Smith

**Affiliations:** 1 Molecular Nutrition Unit, School of Exercise and Nutrition Sciences, Deakin UniversityMelbourne, Australia; 2 Basic and Clinical Myology Laboratory, Department of Physiology, University of MelbourneMelbourne, Australia; 3 School of Health Sciences, University of South AustraliaAdelaide, Australia; 4 Alliance for Research in Exercise, Nutrition and Activity (ARENA), School of Health Sciences and the Sansom Institute for Health Research, University of South AustraliaAdelaide, South Australia; 5 Liggins Institute, University of AucklandAuckland, New Zealand

**Keywords:** Acute exercise, myogenic regulatory factors, resistance exercise training

## Abstract

To enable dynamic regulation of muscle mass and myofiber repair following injury, a satellite cell precursor population exists to supply additional nuclei. Activated satellite cells express many genes and associated proteins necessary for maturation and incorporation into the damaged fiber. There is little knowledge about the response of these markers following whole-body resistance exercise training. We investigated the impact of 12 weeks of progressive whole-body resistance training on the expression of MRFs, PAX7, NCAM, and FA1, incorporating both acute and chronic resistance exercise components. Ten young recreationally active males (21.2 ± 3.5 years) performed 12 weeks of whole-body resistance training at 70–85% of their predetermined one-repetition maximum (1RM). At the initiation and completion of the training period, muscular strength was assessed by RM and dynamometer testing, and vastus lateralis samples were obtained prior to and 3 h following an acute resistance exercise test (both whole-body and isometric exercises). Increased mRNA expression of *PAX7* (threefold), *NCAM* (threefold), *MYF5* (threefold), *MYOD* (threefold) and *MYOGENIN* (twofold) was observed 3 h after the acute resistance exercise test, both pre and posttraining. Similarly, PAX7 (11-fold) and FA1 (twofold) protein abundance increased after acute exercise, while resting NCAM (eightfold) and FA1 (threefold) protein abundance increased following 12 weeks of resistance training. It is possible that these molecular changes are primarily due to the preceding exercise bout, and are not modified by long-term or whole-body exercise training.

## Introduction

Skeletal muscle is dynamically regulated by changes in physical demands and environmental challenges. Exercise training protocols exploit this and lead to increased muscle size and strength gains (Verdijk et al. 2009). Central to this response is the activation of satellite cells; myogenic precursor cells (MPC) responsible for postnatal skeletal muscle growth. A family of Myogenic Regulatory Factors (MRFs: MYF5, MYOD, MYOGENIN, and MYF6) exists to manage MPC fate and facilitate muscle repair. Although the need for MPC involvement in muscle hypertrophy in response to loading (e.g., exercise) is not well defined, resistance exercise training has proved to be very effective in activating MPCs in both younger (Mackey et al. 2011; Bellamy et al. 2014; Farup et al. 2014; Hyldahl et al. 2014) and older adults (Mackey et al. 2007; Verney et al. 2008; Walker et al. 2012; Suetta et al. 2013), and MRFs are also responsive for exercise stimuli (Bickel et al. 2005; Liu et al. 2008; Wilborn et al. 2009; Lindstrom et al. 2010; Snijders et al. 2012; Della Gatta et al. 2014).

The coordination of MRFs and other molecules is important for skeletal muscle adaptation and regeneration, particularly following exercise. Aside from MRFs, several other molecules are commonly used for detecting MPCs and identifying their activation status, namely PAX7 and NCAM. PAX7 is expressed during quiescence and activation of MPCs (Ten Broek et al. 2010) while NCAM is expressed during proliferation and myoblast maturation (Peck and Walsh 1993; Ishido et al. 2006). Recent works have characterized new markers to identify MPCs in skeletal muscle including fetal antigen-1 (FA1, also known as DLK1) (Floridon et al. 2000; Andersen et al. 2009; Waddell et al. 2010). FA1 is important for muscle development and regeneration and is highly expressed in undifferentiated cells of neonatal and fetal skeletal muscle fibers, while in adult tissue, FA1 expressing cells promote regeneration and represent cells with regenerative capacity (Floridon et al. 2000; Waddell et al. 2010). It is unknown how FA1 responds to progressive resistance training protocols.

While there are many studies investigating the molecular markers related to MPC activity following resistance exercise (Crameri et al. 2004; Bickel et al. 2005; Raue et al. 2006; O'Reilly et al. 2008; McKay et al. 2009; Wilborn et al. 2009; Walker et al. 2012), there is less known about their response to resistance training protocols (Mackey et al. 2007, 2009, 2011; Verney et al. 2008; Bellamy et al. 2014; Hyldahl et al. 2014), particularly FA1. Therefore, the aim of this study was to investigate the impact of 12 weeks of progressive whole-body resistance training on the molecular markers, MRFs (MYF5, MYOD, and MYOGENIN), PAX7, NCAM, and FA1, incorporating both acute and chronic resistance exercise components. To bridge the gap in the current knowledge surrounding the temporal expression of these markers and their response to progressive resistance exercise training, biopsies were taken at rest and 3 h following an acute resistance exercise test, at the beginning and end of the program. It was hypothesized that the expression of the MRFs, PAX7, NCAM, and FA1 would increase following acute resistance exercise to indicate a rapid initiation of the myofiber repair process. In addition, it was further hypothesized that there would be an upregulation of NCAM and FA1 in the resting muscle following 12 weeks of whole-body resistance training, which may represent maturing muscle cells used to facilitate skeletal muscle remodelling following exercise-induced adaptation.

## Materials and Methods

### Ethical approval

The resistance training, the measurement of body compositions, and the collection of biopsy samples were undertaken at the University of South Australia and all the procedures were formally approved by the University of South Australia Human Research Ethics Committee and conform to the standards set by the Declaration of Helsinki. Informed written consent was obtained from each subject before the participation in the study, and after the nature, purpose, and risks of the study were explained.

### Participants

Ten healthy young males (21.2 ± 3.5 years [mean ± SD], 1.8 ± 0.09 m, 75.4 ± 7.6 kg) volunteered and provided informed consent to participate in this study. Inclusion criteria required the participants to be aged between 18 and 30 years old, nonsmokers, and recreationally (nonsupervised) resistance trained for ≥6 months (between 2 and 3 sessions per week) prior to the trial commencement. Exclusion criteria included a history of medical condition/illness, or a musculoskeletal injury that would jeopardize participant's safety throughout the trial, or the use of performance enhancing supplements within the previous 3 months. Participants were excluded if they were an elite athlete, currently preparing for or participating at either a national or an international level in any sport. Each volunteer was required to complete the Sports Medicine Australia preexercise screening system (Sports Medicine Australia, 2005), and were only included if deemed apparently healthy and not requiring medical clearance.

### Body composition measures

The body composition was assessed at Weeks 0 and 12, using dual-energy X-ray absorptiometry (DEXA, Lunar Prodigy, General Electric, Madison, WI), using enCORE 2003 software (version 7.52.002). Whole-body composition and nonbone lean tissue mass were determined.

### Exercise protocol

#### 12-week resistance training program

Prior to the trial commencement, each participant was provided with a detailed instruction (by demonstration and coaching) on how to perform all resistance exercises, with participants required to perform each exercise with proper form. Four to seven days later, using the testing protocols recommended by the American College of Sports Medicine (American College of Sports Medicine et al., 2006), one-repetition maximum (1RM) testing was completed for the bench press, hack squat, lat pull down, incline leg press, weighted dip, and biceps curl exercises. Eight-repetition maximum (8RM) testing was completed (American College of Sports Medicine et al., 2006), for the seated calf raise and the seated row exercises. These exercises were then performed throughout the resistance training and the acute bouts of resistance exercise. The results from preliminary testing were used to determine 80% of the participants’ 1RM and 100% of the participants’ 8RM, which was the initial load prescribed at the commencement of training. Test and retest systematic errors were very small (average standardized change in means ±95% CI: 0.08 ± 0.07), with small random errors (average typical error ±95% CI: 0.17 ± 0.12) and nearly perfect test–retest correlations (average ICC ±95% CI: 0.94 ± 0.07).

The whole-body progressive resistance training program was designed, using the recommendations from the American College of Sports Medicine position stand on resistance training in young healthy adults (Ratamess et al. 2009). On training days, participants were required to be present at one of the three testing times (0800–1100, 1200–1500, or 1500–1800) and this time was held constant across all testing. The training program occurred 3 days a week with a minimum of 48 h rest between the sessions. A 10-min warm-up consisting of mobilization exercises (a series of body-weight/unloaded mobilization exercises: walking lunge, a 4 point kneeling hip circumduction, knee fallouts, and a 3-point kneeling stretch over a stability ball) was performed at the commencement of each session. The training consisted of three sets of 8–12 repetitions for each of the aforementioned exercises. Training loads were adjusted throughout the program by the participant such that a maximum of 12 repetitions and a minimum of eight repetitions were completed successfully. Once 12 repetitions could be completed successfully for each of the three sets, the resistance was increased so that only eight repetitions could be completed. In this way, the resistance remained progressive throughout the study. During each training session, a 1-min rest between sets and a 2-min rest between exercises were completed.

#### Single bout of acute resistance exercise

Subjects were required to be 8 h fasted (overnight fasted if presenting at 0800 hours) and euhydrated, and to have not undertaken vigorous physical activity 48 h prior to testing. Two testing days of acute exercise were performed at Day 1 and Week 12 of the training period following a protocol similar to the training days. The aforementioned rest periods between sets and exercises were identical to those allowed during training. Following the same warm-up, each exercise was performed in the same order, with the exclusion of seated calf raise, doing three sets of 8–12 repetitions. The exercises included bench press, hack squat, lat pull down, incline leg press, weighted dip, biceps curl, and seated row. Calf raises were not performed after biopsy at week 0 and 12 as the thigh padding for the seated calf raise station lay directly over the biopsy site; we perceived this to likely cause unnecessary discomfort to the participant.

Maximal isometric torque of the right knee extensors was assessed, using a Biodex System 4 isokinetic dynamometer (Biodex Medical Systems Inc, Shirley, NY). This testing occurred immediately prior to the resistance exercise performed on Day 1 and at Week 12 referred to above. Participants were positioned on the dynamometer with their knee joint flexed to 90° and the axis of rotation of the knee joint aligned with the axis of rotation of the lever arm of the dynamometer. The lever arm of the dynamometer was strapped to the participants’ ankle at 3 cm above the medial malleolus. The seat position data were recorded to enable replication of the position for subsequent testing. Three submaximal warm-up efforts of 5-sec duration were performed with a 1-min rest between efforts. After a 2-min rest, three maximal 5-sec isometric efforts were performed with a 1-min rest between efforts. Participants were vigorously verbally motivated during each maximal effort.

#### Muscle biopsy procedure

Tissue samples were harvested from the vastus lateralis of the dominant leg under local anesthesia (lignocaine 1%) by percutaneous needle biopsy technique (Bergstrom 1975) modified to include suction (Evans et al. 1982). The tissue was sampled prior to and 3 h following the acute resistance exercise test (whole-body and isometric testing) that was performed at the beginning and conclusion of the 12-week training period (48–72 h since last training session). As a result, the volume of exercise that the postresistance exercise tissue sample had been exposed to was equivalent to a standard training day plus the additional isometric testing referred to above. To avoid additional regional inflammation, the biopsy needle was angled away inferiorly and medially to avoid the initial biopsy site. Following the biopsy, samples were immediately frozen in liquid nitrogen stored in −80°C for later analysis.

#### Total RNA extraction and qRT-PCR

RNA was extracted, using the ToTALLY RNA™ Total RNA Isolation Kit according to manufacturer's instructions (Life Technologies, Victoria, Australia) from 5 to 10 mg of skeletal muscle as previously described (Trenerry et al. 2007). RNA quality and concentration were determined, using the NanoDrop 1000 (Thermo-Fisher Scientific, Australia). First-strand cDNA was generated, using 0.5 *μ*g of the total RNA using the High-Capacity RNA-to-cDNA™ Kit according to the manufacturer's instructions (Life Technologies). qRT-PCR was performed in triplicate using the ABI 7500 sequence detection system (Life Technologies), with reactions containing Power SYBR® Green PCR Master Mix (Life Technologies), forward and reverse primers and cDNA template (1.25 ng/*μ*L). RT-positive and RT-negative controls as well as nontemplate controls were also performed for each primer set. *GAPDH* gene expression showed no change in response to exercise and was, therefore, used for normalization of the target genes. Data were analyzed using a comparative quantification cycle (Cq) method where the amount of target was normalized to the amount of endogenous control, relative to control value given by 2^−ΔΔCq^. Data were then normalized to the pretraining baseline values. Primers were designed using Primer Express v3 (Applied Biosystems, Foster City, CA) spanning intron-exon boundaries to prevent amplification of the target region for any contaminating DNA. Primer sequences are listed below (see Table 1) or have been described previously (Trenerry et al. 2007). Primer sequence specificity was also confirmed using Basic Local Alignment Search Tool (BLAST). A melting point dissociation curve was generated by the PCR instrument for all PCR products to confirm the presence of a single amplified product.

**Table 1:** Details of primers used for qRT-PCR analysis

Gene	GenBank accession number	Forward primer (5′–3′)	Reverse primer (5′–3′)
*FA1*	NM_003836	GGACGATGGCCTCTATGAATG	TCCTTTTTCTGGCAGTCCTTTC
*MYF5*	NM_005593	TTCTACGACGGCTCCTGCATA	CCACTCGCGGCACAAACT
*MYOD*	NM_002478	CCGCCTGAGCAAAGTAAATGA	GCAACCGCTGGTTTGGATT
*MYOGENIN*	NM_002479	GGTGCCCAGCGAATGC	TGATGCTGTCCACGATCGA
*NCAM*	NM_000615	GAAAAGCTCACCCCAAACCA	GGGTGGAGGAGGAATCATCAT
*PAX7*	NM_002584	GCCACAGCTTCTCCAGCTACTC	TGACCGGGTTCATGTGGTT

Primer sequences were designed using Primer Express Software v3.0 (Applied Biosystems) using sequences accessed through GenBank and checked for specificity using nucleotide-nucleotide BLAST search.

*FA1*, fetal antigen-1; *NCAM*, neural cell adhesion molecule.

#### Protein extraction and immunoblotting

Skeletal muscle (10 mg) was prepared as described previously (Caldow et al. 2013). Proteins (25 *μ*g) were separated by 10% SDS–PAGE and transferred to nitrocellulose membranes (Bio-Rad Laboratories) via wet transfer. The membranes were blocked in either 5% w/v Bovine Albumin Serum (BSA) (Sigma, St Louis, MO) in Tris buffered saline with 0.1% Tween 20 (TBST) or 5% (w/v) skim milk powder (SKIM) in TBST for 2 h at room temperature. Where possible, membranes were cut according to size to allow several antibodies to be blotted on a single membrane. Membranes were incubated in primary antibodies diluted in blocking buffer at 4°C overnight: actin (#A2066; BSA, Sigma-Aldrich), Fa1 (#2069; BSA, Cell Signaling, Danvers MA), NCAM (123C3) (#sc-7326; BSA, Santa Cruz Biotechnology), and Pax7 (ARP32742_9050; SKIM, Aviva Systems Biology, San Diego, CA). Following subsequent washes with TBST, the membranes were incubated with appropriate horse radish peroxidase-conjugated secondary antibodies at room temperature for 1 h prior to further washes. Proteins were visualized by enhanced chemiluminescence (Perkin Elmer Lifesciences, Boston, MA) using Kodak Image Station (Model: 440CF, Eastman Kodak Company) and quantified by densitometry software (Kodak 1D 3.5). An appropriately sized membrane section was stripped (Restore™ Western Blot Stripping Buffer; Thermo-Fisher Scientific) and reprobed for actin. Actin was used as a loading control because its protein abundance was not changed with acute exercise or training. Raw densitometry values for FA1, NCAM, and Pax7 were normalized to actin (NB: NCAM and FA1 were on the same blot, therefore used the same actin). Data were then normalized to pretraining baseline values.

### Statistical analysis

Statistical analysis was performed using the statistical software package SPSS 14.0 (Chicago). Data were not different from a normal distribution as determined using a Shapiro–Wilkes test. Two-way analysis of variance with repeated measures was used to evaluate effects of the timing of the assessments in terms of the training program (pretraining and posttraining) and the pre- and postacute exercise sessions on the markers of satellite cell activity. Paired *t*-tests were used to compare baseline and Week 12 means for strength and body composition. The sequential Bonferroni procedure was used to correct for multiple comparisons for all probabilities associated with the t-test. Data are presented as mean ± standard error of mean (SEM) or standard deviation (SD) where specified. The probability level of <0.05 was adopted throughout to determine statistical significance. A probability level of <0.1 was adopted to indicate a trend for statistical significance.

## Results

### Strength increased following 12 weeks of resistance training

Subject compliance to the resistance training program was monitored via training diaries and was very high (91% compliance or 3/36 missed training sessions/subject across 12 weeks). Resistance training significantly increased lower limb strength for squat (26%, *P *<* *0.001), incline leg press (34%, *P *<* *0.001), calf raise (52%, *P *<* *0.001), and maximal isometric torque (7%, *P* < 0.01). Upper limb strength increased for the bench press (4%, *P *<* *0.01), lat pull down (11%, *P *<* *0.001), weighted dip (8%, *P *<* *0.01), bicep curl (5%, *P *<* *0.05), and seated row (21%, *P *<* *0.001) following training. Total tissue increased (*P* < 0.05) while there was an increasing trend in lean tissue mass (*P* = 0.059) (Table 2). Fat mass remained unchanged.

**Table 2:** Strength and body composition changes in response to 12 weeks of resistance training: comparison of untrained and trained states

	Baseline	Post-training
Exercise (1RM)		
Bench press (kg)	86.8 ± 15.5	90.6 ± 11.3**
Squat (kg)	108.5 ± 18.1	137.1 ± 13.2**
Incline leg press (kg)	279.0 ± 27.5	398.0 ± 42.4***
Lat pull down (kg)	63.5 ± 8.6	70.5 ± 9.0***
Weighted dip (kg)	114.9 ± 12.7	123.5 ± 12.8**
Bicep curl (kg)	46.0 ± 6.7	48.3 ± 6.7*
Seated calf raise (kg)^	40.3 ± 9.9	61.5 ± 14.3***
Seated row (kg)^	41.3 ± 7.7	49.8 ± 7.2***
Max isometric knee extension torque (N·nm)#	290.1 ± 61.9	311.1 ± 55.5
DEXA measure		
Total tissue (g)	74614 ± 7362	76245 ± 7295*
Lean tissue (g)	59601 ± 5178	60678 ± 5617†
Fat tissue (g)	11673 ± 3935	12235 ± 4444

Before and after training, participants performed 1RM testing (^ indicates 8RM for this exercise) and an isometric test on an isokinetic dynamometer (^#^) as a measure of their strength. Participants also underwent a DEXA scan to measure body composition before and after training. Values are presented as means ± SD of 10 young men.

* *P* < 0.05

** *P* < 0.01

*** *P* < 0.001 indicates a difference between baseline and post-training.

† *P* < 0.1 indicates a trend for a difference between baseline and post-training.

### Myogenic markers were significantly increased in response to acute resistance exercise bouts

**Figure 1:**
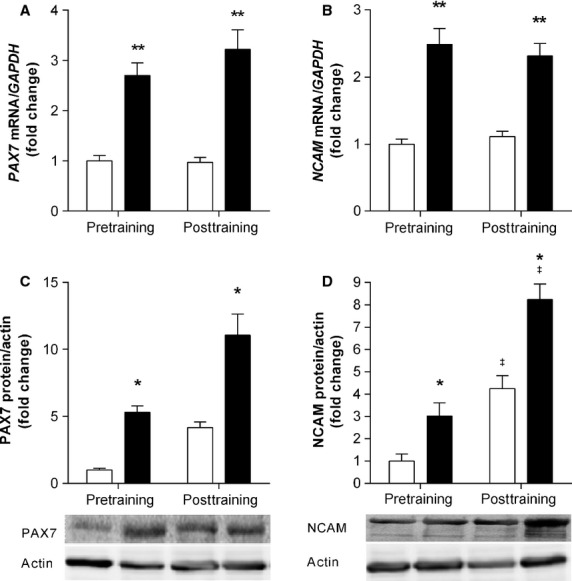
mRNA and protein expression of markers associated with satellite cell activation change following acute resistance exercise, irrespective of training. mRNA and protein expression of *PAX7* (A, C) and *NCAM* (B, D) were measured before and after 12 weeks of resistance training. Open bars (□) represent resting; closed bars (▪) represent 3 h postexercise. mRNA values were normalized to *GAPDH* and protein to Actin, representing the mean ± SEM of 10 young men. All values are presented as fold change, normalized to pretraining baseline values. The sum of both phosphorylated NCAM bands was taken. *denotes statistical significant effect for an acute resistance exercise bout (*P *<* *0.05), **P < 0.01. ^‡^denotes a statistically significant training effect between pre- and posttraining (*P *<* *0.05).

Baseline mRNA expression of *PAX7* and *NCAM* were the same before and after 12 weeks of training (Fig. 1A and B). In response to resistance exercise, *PAX7* and *NCAM* mRNA increased approximately threefold (main effect for acute exercise; *P *<* *0.001 and *P *<* *0.01, respectively) in the pre- and posttraining samples. Protein expression also increased following acute exercise (main effect for acute exercise). PAX7 protein was highly expressed 3 h post resistance exercise, both before (fivefold, *P *<* *0.05) and after (11-fold, *P *<* *0.05) resistance training. The fourfold increase at rest following training was not statistically different to those at baseline (Fig. 1C). NCAM expression was higher following resistance training both at rest (main effect for training; fourfold, *P *<* *0.05) and after acute resistance exercise (main effect for acute exercise; eightfold, *P *<* *0.05) (Fig. 1D).

**Figure 2:**
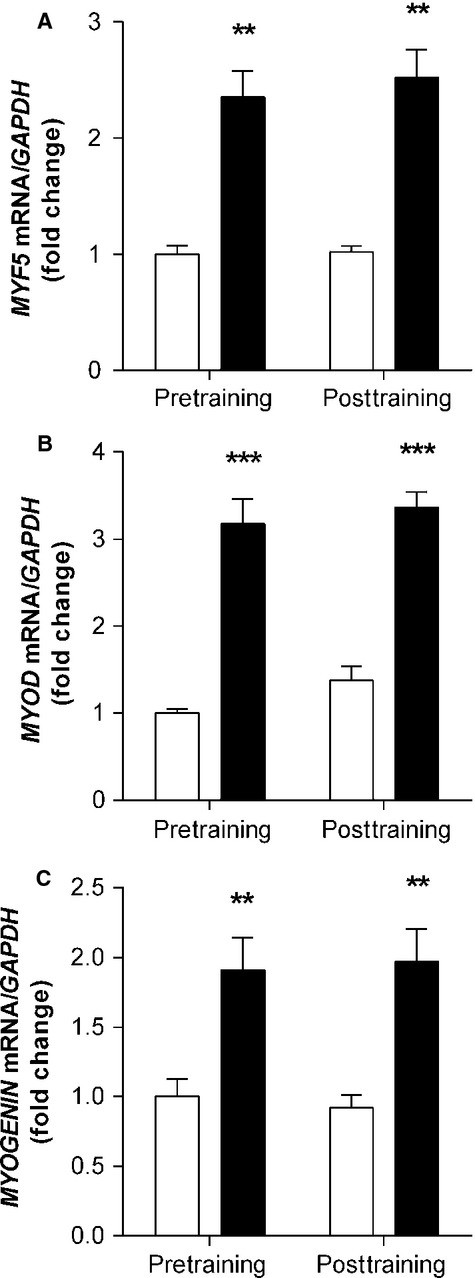
Myogenic regulatory factors responsible for satellite cell activation, proliferation and differentiation are significantly increased in response to acute resistance exercise, but not influenced by training. mRNA expression of MRFs *MYF5, MYOD,* and *MYOGENIN* were measured before and after 12 weeks of resistance training. Open bars (□) represent resting; closed bars (▪) represent 3 h postexercise. mRNA values were normalized to *GAPDH*, representing the mean ± SEM of 10 young men. All values are presented as fold change, normalized to pretraining baseline values. **denotes statistical significant effect for an acute resistance exercise bout (*P *<* *0.01), ***P < 0.001.

Concomitant increases in *MYF5* (2.5-fold, *P *<* *0.01), *MYOD* (threefold, *P *<* *0.001), and *MYOGENIN* (twofold, *P* < 0.01) mRNA were observed in response to acute exercise in both the pre- and posttraining samples (main effect for acute exercise). Resistance training did not alter mRNA expression as elevations following resistance exercise were of a similar magnitude to those before training, and there were no differences in the resting samples (Fig. 2).

### FA1, a marker of satellite cells with regenerative potential, is responsive to exercise training

**Figure 3:**
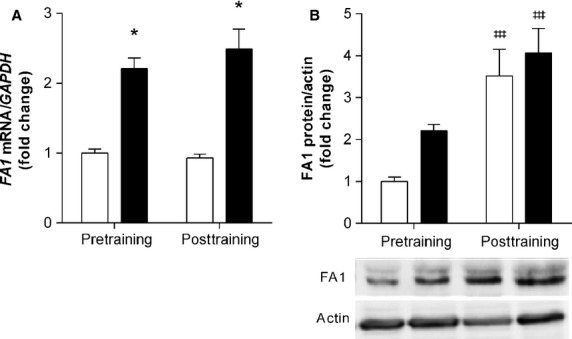
Following acute resistance exercise and training there are changes in the expression of *FA1*, a marker of satellite cells with regenerative potential. mRNA and protein expression of *FA1* (A, B) were measured before and after 12 weeks of resistance training. Open bars (□) represent resting; closed bars (▪) represent 3 h post-exercise. mRNA values were normalized to *GAPDH* and protein to Actin, representing the mean ± SEM of 10 young men. All values are presented as fold change, normalized to pretraining baseline values. ^‡‡‡^denotes a statistically significant training effect between pre- and posttraining (*P *<* *0.001).

*FA1* mRNA was upregulated 3 h following resistance exercise (Fig. 3A) both before and after resistance training (main effect for acute exercise; 2.5-fold, *P *<* *0.05). Similar to NCAM, FA1 protein was elevated at rest in response to resistance exercise following 12 weeks of resistance training (main effect for training; 3.5 fold, *P *<* *0.001), compared to pre-training values (Fig. 3B).

## Discussion

In response to exercise-induced trauma, skeletal muscle has the ability to regenerate and remodel because a pool of quiescent MPCs exists. In the present study, the impact of 12 weeks of resistance training on the markers of satellite cell activation and proliferation was examined in recreationally active males, with samples obtained at rest and 3 h after an acute resistance exercise test. The main findings from this study were the increases in FA1 and NCAM protein expression following the resistance training, as well as the consistent elevation in *PAX7, NCAM, FA1, MYF5, MYOD,* and *MYOGENIN* mRNA expression within 3 h of resistance exercise in post-training samples.

In the present study, it would appear that the satellite cell pool retains its “responsiveness” to acute resistance exercise even though the muscle has been through progressive resistance training for 12 weeks, as acute exercise tests before and after 12 weeks of training resulted in similar magnitude increases in *PAX7, NCAM, MYF5, MYOD,* and *MYOGENIN* mRNA expression. Upstream myokine induced-signaling pathways involved with the regulation of satellite cell proliferation, namely IL-6 (Croisier et al. 1999; Willoughby et al. 2003; McKay et al. 2009; Trenerry et al. 2011) and STAT3 (Trenerry et al. 2011) are also equivalent after several weeks of resistance training. This suggests that the acute exercise response of MRFs, NCAM, and PAX7 is not modified by long-term and whole-body exercise training, but rather may be primarily responsive to the preceding exercise bout. Studies are required to examine the specificity of “responsiveness” to factors such as total muscular load and eccentric/concentric loading (Mackey et al. 2011; Joanisse et al. 2013).

The concomitant expression of PAX7, MYF5, MYOD, and NCAM may suggest MPC activation, cell cycle entry and proliferation following resistance exercise (Ishido et al. 2004, 2006; Mackey et al. 2009; Singh and Dilworth 2013); facilitating muscle remodelling within the early critical hours of exercise recovery. Previous research has demonstrated an exercise-induced activation (and maintained elevation) of the aforementioned markers that may last for several days following an exercise bout (Psilander et al. 2003; Crameri et al. 2004; Bickel et al. 2005; O'Reilly et al. 2008; Mackey et al. 2009; Bellamy et al. 2014; Hyldahl et al. 2014). Of the few studies that have measured satellite cell activity immediately following resistance exercise they observed comparative exercise-induced elevations (˜1.5–5-fold) in MRFs and PAX7 within the early hours of exercise recovery (Willoughby and Nelson 2002; Psilander et al. 2003; Raue et al. 2006; McKay et al. 2009; Deldicque et al. 2010; Walker et al. 2012). Satellite cell activation is an important step in the myogenic program and the repair and regeneration process. We have demonstrated that activation can occur within 3 h of a resistance exercise bout, further highlighting the rapid and dynamic nature of muscle adaptation.

Elevations in *MYOGENIN* and NCAM mRNA indicate that a pool of maturing satellite cells exists, with the arrest of the cell cycle, an increase in satellite cell differentiation and a commitment to the myogenic lineage. This has previously been observed for more than 12 h following resistance exercise (Bickel et al. 2005). The presence of NCAM also represents myoblasts that are maturing and beginning to fuse to existing fibers (not only active satellite cells) (Peck and Walsh 1993). *MYOGENIN* was twofold higher following acute resistance exercise compared with resting samples. Other studies have observed similar elevations in myogenin at 0.5, 2, 4, 6, 8 and 12 h (Willoughby and Nelson 2002; Psilander et al. 2003; Yang et al. 2005; Raue et al. 2006; Wilborn et al. 2009; Deldicque et al. 2010) post resistance exercise. It has also been suggested that when MYOGENIN expression is elevated immediately following an insult such as functional overload, it may represent a subset of MPCs immediately ready for differentiation with little or no proliferation as it seems unlikely that these cells have progressed through the cell cycle so quickly (Ishido et al. 2004).

FA1 has been used both as a marker of proliferation and as a marker to identify cells that are destined for regeneration and repair (Floridon et al. 2000; Crameri et al. 2004; Waddell et al. 2010). The upregulation of FA1 following 12 weeks of training may be indicative of muscle cells that are undergoing maturation and facilitating regeneration (Floridon et al. 2000; Andersen et al. 2009; Waddell et al. 2010; Snijders et al. 2012), similar to NCAM (Peck and Walsh 1993). In response to a single bout of high-intensity exercise, FA1 has been elevated for up to 8 days (Crameri et al. 2004; Snijders et al. 2012); the present study is the first to examine FA1 in the early hours of resistance exercise recovery, highlighting that there are cells present destined for muscle regeneration.

The molecular markers examined in the current study are indirect measures of MPC activity. However, these proteins are used frequently to determine the abundance and status of MPCs. It cannot be overlooked that the upregulation of FA1 may indicate other exercise-responsive cell populations, as there are many cell types present in skeletal muscle such as fibroblasts and inflammatory cells, which also express FA1 (Andersen et al. 2009; Waddell et al. 2010). Interestingly, it is thought that FA1 from neighboring cells interacts with MPCs to influence myofiber regeneration (Andersen et al. 2009; Waddell et al. 2010).

Commonly used molecular markers of satellite cell activation may be responsive to the mechanical loading associated with resistance exercise during the critical recovery window. A rapid activation of satellite cell-specific transcription factors (MYF5, MYOD and MYOGENIN) and membrane proteins (PAX7, NCAM and FA1) was observed within 3 h of resistance exercise completion. This may indicate that as early as 3 h into the recovery period, the myofiber repair process has been initiated by satellite cell activation. With 12 weeks of resistance training, satellite cells are proliferating, maturing, and potentially fusing to repair and increase the size of the myofiber, suggesting that this mode of exercise may be sufficient to induce skeletal muscle remodelling. Additionally, it is possible that the satellite cell pool retains its “responsiveness” to acute resistance exercise even though the muscle has been through progressive resistance training for 12 weeks, as indicated by similar magnitude increases in satellite cell-specific transcription factors and membrane proteins following the second exercise test (at week 12). This study is, however, confounded by the lack of data providing immunolocalization of the measured markers as this analysis could not be performed in this study due to sample cryodamage. Yet the specificity of many of the measured transcription factors in mediating myogenic programming, cellular proliferation, and maturation is well established.

Strength increases elicited by the training program varied by exercise, with upper body exercises using smaller muscle groups (i.e. bench press, bicep curl) eliciting the smallest gains and exercises using larger amounts of muscle mass resulting in larger strength gains (i.e. squat, leg press). This response is unsurprising given that multi-joint exercises utilizing large amounts of muscle mass are recommended for positive strength adaptations (Stone et al. 1998; Ratamess et al. 2009). The magnitude of strength gains observed in the lower body strength measures indicate that although subjects were recreationally trained, their capacity for adaptation at the muscular and (given the data presented above regarding FA1 and various MRF's) molecular level was not exhausted. These data collectively provide evidence for the ongoing adaptive processes and the outcomes present in trainees who are not totally naive to resistance training.

The results from this study contribute to the growing body of knowledge surrounding the rapid satellite cell response to acute resistance exercise and training; however, they only provide a snapshot of what is occurring. Indeed these data are consistent with the emergent evidence that satellite cell activation, measured by changes in the number of cells, is increased at 24 h after resistance exercise (Bellamy et al. 2014; Farup et al. 2014; Hyldahl et al. 2014). This study demonstrates that the rapid and robust molecular adaptations that occur within the first few hours after acute resistance exersise may contribute to this phenotypic adaptation and these changes are not modified by long- term and whole-body exercise training, but rather are primarily responsive to the preceding exercise bout. Expression studies on cells other than satellite cells, including mature myofibers, would provide further insights into the coordinated myogenic programming necessary to facilitate global muscular adaptation and regeneration.
